# Supplementary material for: Pseudomonas putida Biofilm Depends on the vWFa-Domain of LapA in Peptides-Containing Growth Medium
Source: Int J Mol Sci. 2022 May 24;23(11):5898. doi: 10.3390/ijms23115898 (PMC9180339; doi:10.3390/ijms23115898)
Supplement: Supplementary file 1 [file ijms-23-05898-s001.zip › ijms-1714847-supplementary/Supplementary Tables.pdf]

Table S1. Oligonucleotides

Vector[1]	Name[2]	Sequence[2]	Purpose
pEMG-lapB-lapA	1.2-EMG5’	GGCAGGTTGGGCGTCGC	For pEMG amplification
	3.1-*lapB3’*-SceI-EMG3’	*GCAACGCTGTTTGAACGTATCG*ATTACCCTGTTATCCCTACGCGAAGTCGCTCTTCTTGATGG	For pEMG amplification
	5.1-*lapB5’*-**DS**-SD- ***lapA5’***	*GGACTCGACTGACTTCGGATTCCAC***ACTTAACCCTTCT**AGAAGGTGGTTGT***ATGAGCAGCGTTG TAGCCATCGT***	For amplification *lapA* 5’ terminus
	2.3.-EMG-SceI-***lapA3’***	GCGACGCCCAACCTGCC ATTACCCTGTTATCCCTA ***GAAATCTGGCCACCGTCGATG***	For amplification of *lapA* 5’ terminus
	4.1-lapB3’	CGATACGTTCAAACAGCGTTGC	For amplification of *lapB* 5’ terminus
	6.1-**lapB5’**	GTGGAATCCGAAGTCAGTCGAGTCC	For amplification of *lapB* 5’ terminus
pEMG-lapB-Pm-xylS-lacI-Ptac-lapA	11-Pm-**DS**-*lapB5’*	CCGCGGTT**AGAAGGGTTAAGT***GTGGAATCCGAAGTCAGTCGAGTCC*	For pEMG-lapB-lapA amplification
	12- Ptac-SD-*lapA5’*	CTAGAGAGAAGGTGGTTGT*ATGAGCAGCGTTGTAGCCATC*	For pEMG-lapB-lapA amplification
	9-T1-xylS-*lacI3'*	TGGGCGCG*TTGCGTTGCGCTCACTGC*	For amplification of *lacI*-P_{tac} cassette; for assessing the integrity of pEMG-PtacA-natB, and sequencing
	10-lapA5’-**DS**-*Ptac*	GCTGCTCAT**ACAACCACCTTCT***CTCTAGAGTCGACCTGCAGGC*	For amplification of *lacI*-P_{tac} cassette
	7-lapB5’-**DS**-*Pm*	ATTCCAC**ACTTAACCCTTCT***AACCGCGGCCTAGGCATG*	For amplification of Pm-*xylS*-T1 cassette
	8-lacI3’**-xylS-T1**	**AGCGCAACGCAA***CGCGCCC AGCTGTCTAGG*	For amplification of Pm-*xylS*-T1 cassette; for assessing the integrity of pEMG-PtacA-natB, and sequencing
pEMG-PtacA-natB	1.3.-(lap)Aprom3’-**T1**	GCCGCTCTGGCAGGGAAGTAC**GGAAGTGGCTTGATTACGAACG**	For pEMG-lapB-Pm-xylS-lacI-Ptac-lapA amplification

	3.3.-(lap)Aprom5’-*EMG3*	AAGTCTGGTGAAAATG*ATTACCCTGTTATCCCTACGCGAAG*	For pEMG-lapB-Pm-xylS-lacI-Ptac-lapA amplification
	13.-(lap)Aprom3’	GTACTTCCCTGCCAGAGCGGC	For amplication of native *lapA-B* promoter area
	14.-*EMG3*-(lap)Aprom5’	*GGGTAAT* CATTTTCACCAGACTTA GCGGG	For amplication of native *lapA-B* promoter area
pGNW-lapA-Avwf$^-$	3.5-A5vwf5-SceI-*EMG3*	ACGTTGGTAGTGGTGCTGCTGATTACCCTGTTATCCCTA*CGCGAAGTCGCTCTTCTTGATGG*	For pEMG amplification
	4.5.-A5vwf5	CAGCAGCACCACTACCAACGT	For amplification of *lapA* 5’ DNA from vWFa domain
	6.5.1.-A3vwf5-*A5vwf3*	CTGATAGTAGGT *GCTATCGACCATGAAGGCGATGTTG*	For amplification of *lapA* 5’ DNA from vWFa domain
	5.5.1.-*A5vwf3*-A3vwf5	*GGTCGATAGC*ACCTACTATCAGAGCAATGAGTCCACC	For amplification of *lapA* 3’ DNA from vWFa domain
	2.5.-*EMG*-SceI-*A3vwf3*	GCGACGCCCAACCTGCCATTACCCTGTTATCCCT*CGTTACCTTTGCCACCATTCAG*	For amplification of *lapA* 3' DNA from vWFa domain
pEMG-ΔlapG	PP0164-I-fw	GAGCTCTTGTTGGCGAACGG	For the construction of *lapG* deletion (PP_0164); and assessment of *lapG* deletion
	PP0164-I-rev	CAGTGACATCTGTGCTCCTGGCAAAGCCGACATAGC	For the construction of *lapG* deletion (PP_0164);
	PP0164-2-fw	AGGAGCACAGATGTCACTG	For the construction of *lapG* deletion (PP_0164);
	PP0164-2-rev	GGCAACTCTGGCAGGCT	For the construction of *lapG* deletion (PP_0164); and assessment of *lapG* deletion
pEMG-ΔlapE	PP4519-I-fw-EcoRI	TTGAATTCGTGACTACCTCAGTGGTG	For the construction of *lapE* deletion (PP_4519); and assessment of pEMG-DlapE integrity and *lapE* deletion

	PP4519-I-rev	CCATTCGCAGCACAAAGCGGGTACATCTCCCTGATCCTG	For the construction of *lapE* deletion (PP_4519)
	PP4519-2-fw	CGCTTTGTGCTGCGAATGGG	For the construction of *lapE* deletion (PP_4519)
	PP4519-2-rev-BamHI	TTGGATCCGGTTATGTGGCAAACTG	For the construction of *lapE* deletion (PP_4519); sequencing
Validation	EMG-traJ	GGCAAGGTCATGATGGGCG	For assessing the integrity of pEMG vectors, and sequencing
	lapB3'-taga	CGGTAGGCCTGTTGTCAGC	For assessing the integrity of pEMG vectors, and sequencing
	lapA-rev	TGGATCCGACAATGCTTTTGACGATGG	For assessing the integrity of pEMG vectors, and sequencing
	lapA4	CGATTGTATCGGTCACTTGGG	For sequencing *lapA* 5' terminus of constructed PANB-stains
	lapB4	CGTAGCCGATGCCCAGAG	For sequencing *lapB* 5' terminus of constructed stains
	lapA-RACE1	AGCTCAGTAGTCGGGTCAA	For assessing the integrity of pEMG vectors, and sequencing
	Prtac	AATTAATCATCGGCTCGTATAA	For assessing the integrity of pEMG vectors, and sequencing
	lacI-K	CAGCTGGCACGACAGGTTTC	For assessing the integrity of pEMG vectors, and sequencing
	xylS-K	CAATGCAAAAAGGAAGTGGCTTG	For assessing the integrity of pEMG-lapB-Pm-xylS-lacI-Ptac-lapA, and sequencing

	XylS-alg	ATGGATTTTTGCTTATTGAACGAG	For assessing the integrity of pEMG-lapB-Pm-xylS-lacI-Ptac-lapA, and sequencing
	xylS-välja	GGGCAGGCGAATAGAGTG	For assessing the integrity of pEMG-lapB-Pm-xylS-lacI-Ptac-lapA, and sequencing
	Pm-suunas	TTCACCAGTTTTGATTTAAACG	For assessing the integrity of pEMG-lapB-Pm-xylS-lacI-Ptac-lapA, and sequencing
	Pm-eemale	GTTTTCCTGGTATACCGCCG	For assessing the integrity of pEMG-lapB-Pm-xylS-lacI-Ptac-lapA, and sequencing
	PP0167-down	TTTGGATCCGCGGCGCTCTCTTCATCC	For sequencing *lapAB* promoter-area of constructed PANB-stains
	Avwf-K2	GAAGGCAACGACATCTTCGTC	For assessing the integrity of pGNW-lapA-Avwf^{-}, and sequencing
	Avwf-K1	AGGGTTGGTGGACTCATTGC	For sequencing *lapA* of constructed Avwf^{-} -stains
	A5vwf-K3	CCAAGGAAGTCTGGACCAGC	For sequencing *lapA* of constructed Avwf^{-} -stains
	A3vwf-K4	CTTGTTGAAGTCCTTGATCACGTC	For sequencing *lapA* of constructed Avwf^{-} -stains
	LapE-fw-BamHI	TGGATCCAACCCTGCACAAGCCAAAG	For sequencing *lapE* flanking DNA, and assessment of pEMG-DlapE integrity and *lapE* deletion

[1] Vector for which the oligonucleotides were used

[2] Underlined text, text in italic or bold indicates the match of element and sequence

Table S2 Used growth media

**Abbreviation**	**Components**
LB	10 g/L tryptone (LabM, Heywood, UK)<br>5 g/L yeast extract (LabM, Heywood, UK)<br>5 g/L NaCl
M9-0.2CAA	M9 buffer [1]<br>2.5 mL/L of microelements [2]<br>2 g/L of glucose<br>2 g/L CAA (casamino acid; Difco, Detroit, MI, USA) and 0.01 g/L tryptophan
M9-0.2CAA + pK	M9-0.2CAA was amended with<br>0.4 mg/ml poly-L-lysine (1000-5000 Da; Sigma-Aldrich, St. Louis, MO, USA)
M9-0.2CAA + tryptone	M9-0.2CAA was amended with<br>10 g/L tryptone (LabM, Heywood, UK)
M9-0.2CAA + cellulose	M9-0.2CAA was amended with<br>0.4 g/L the sodium salt of carboxyl methylcellulose (Sigma-Aldrich, St. Louis, MO, USA)
M9-0.2CAA + PGA	M9-0.2CAA was amended with<br>PGA (poly-galacturonic acid)

1. Adams, M.H. *Bacteriophages*; Interscience Publishers, Inc.: New York, N.Y., 1959.
2. Bauchop, T.; Elsden, S.R. The Growth of Micro-organisms in Relation to their Energy Supply. *Microbiology (Reading, England)* **1960**, *23*, 457-469, doi:https://doi.org/10.1099/00221287-23-3-457.
