# Supplementary figures and images for: Pseudomonas putida Biofilm Depends on the vWFa-Domain of LapA in Peptides-Containing Growth Medium
Source: Int J Mol Sci. 2022 May 24;23(11):5898. doi: 10.3390/ijms23115898 (PMC9180339; doi:10.3390/ijms23115898)

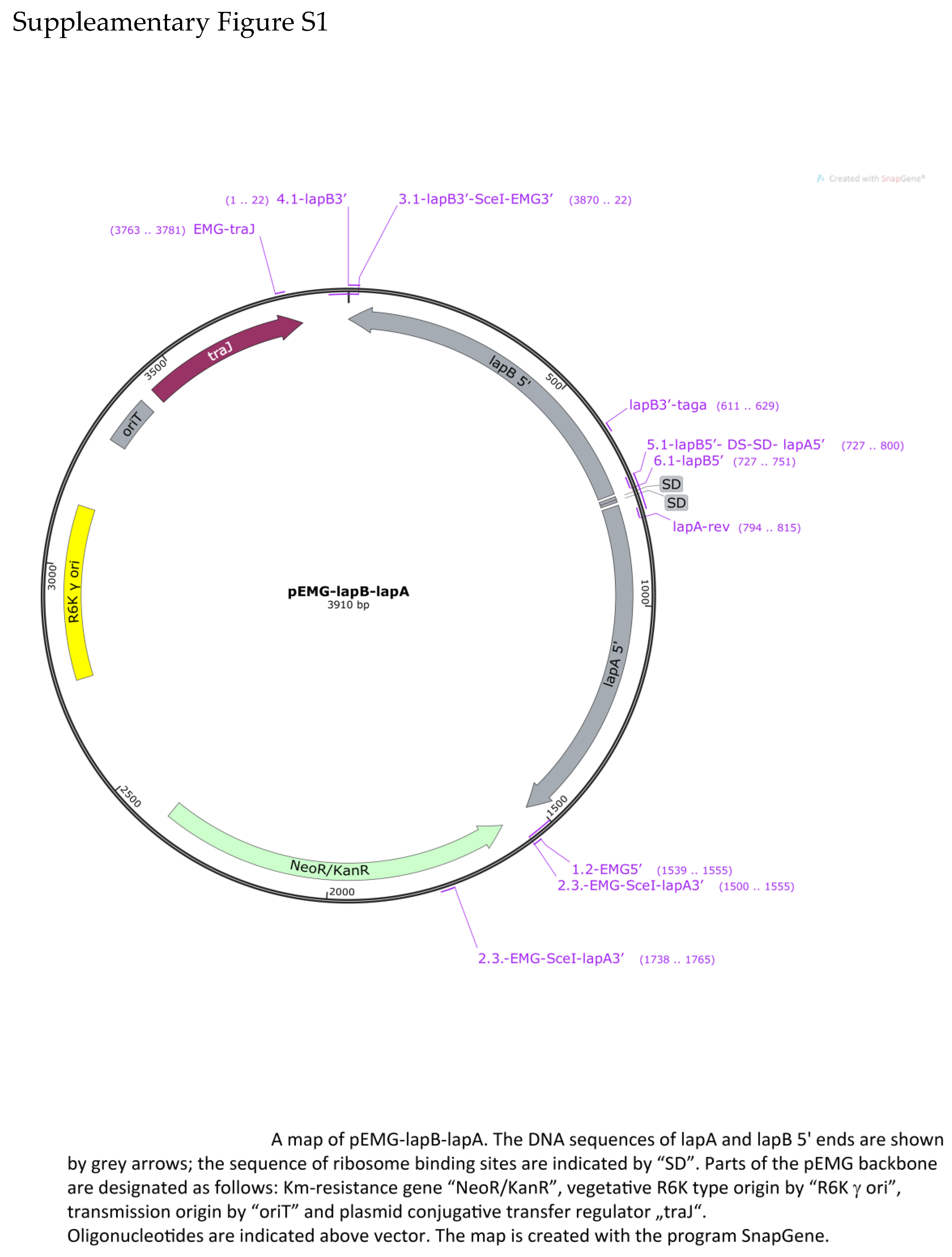

Supplement: Supplementary file 1 [file ijms-23-05898-s001.zip › ijms-1714847-supplementary/Supplementary Figure 1.tif]

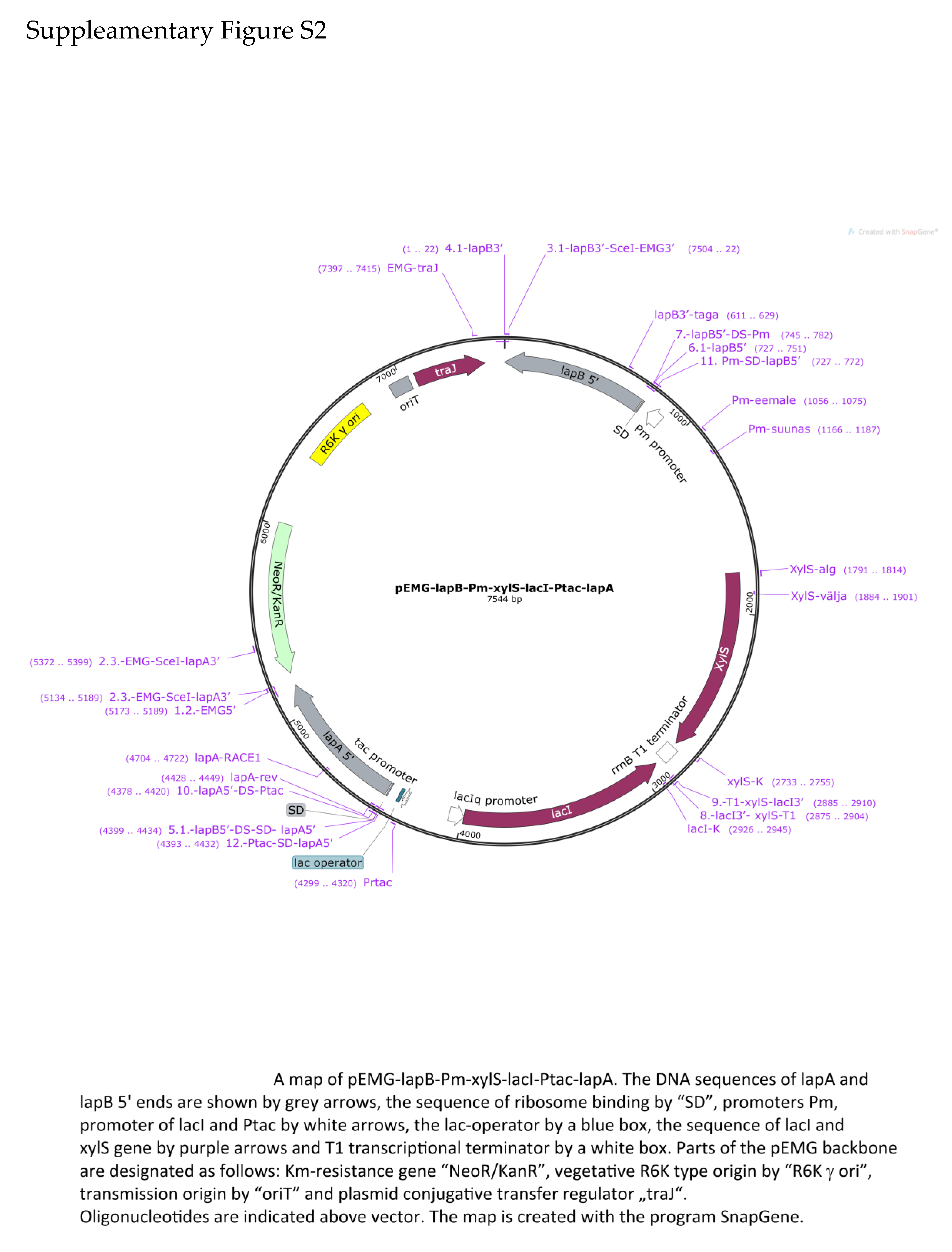

Supplement: Supplementary file 1 [file ijms-23-05898-s001.zip › ijms-1714847-supplementary/Supplementary Figure 2.tif]

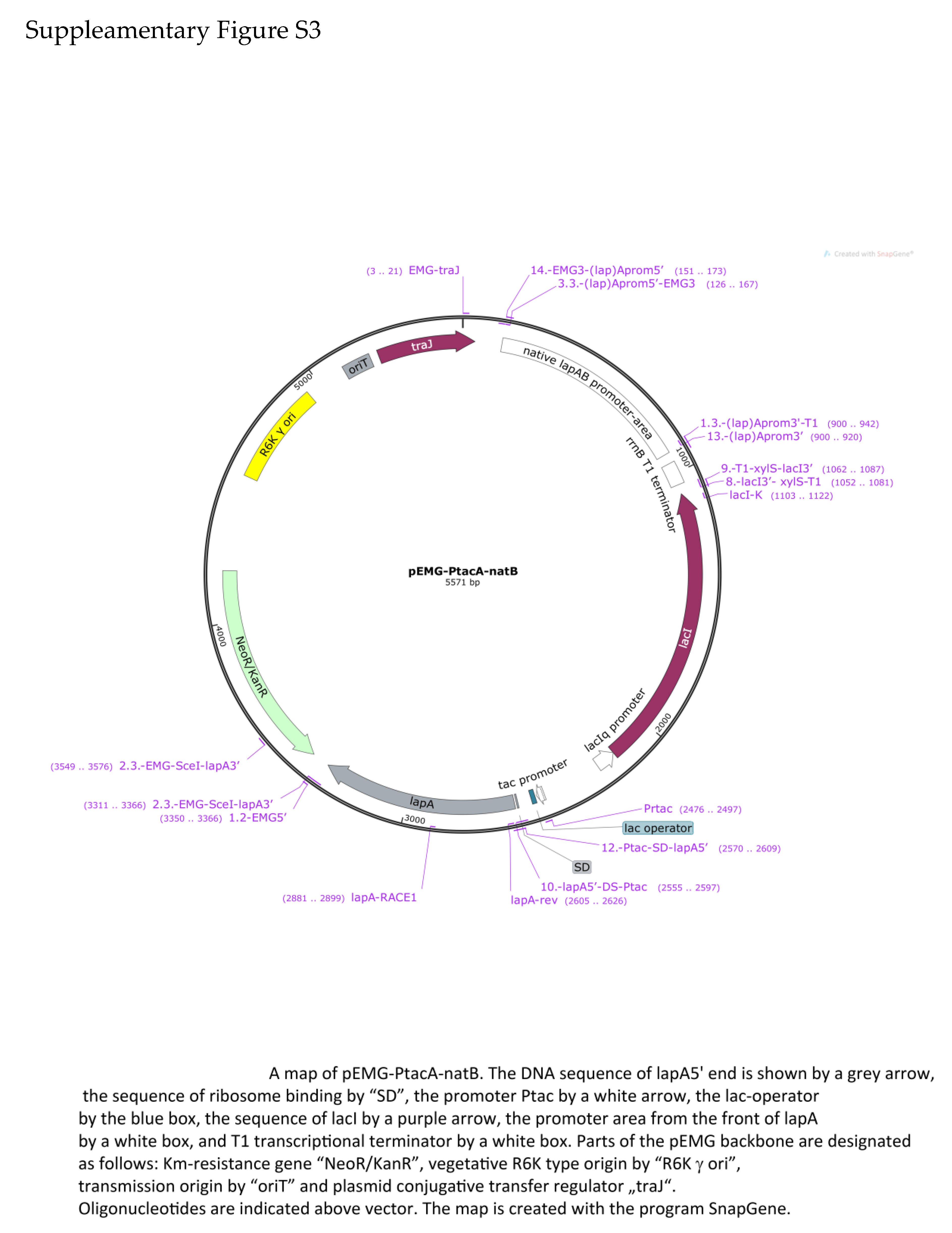

Supplement: Supplementary file 1 [file ijms-23-05898-s001.zip › ijms-1714847-supplementary/Supplementary Figure 3.tif]

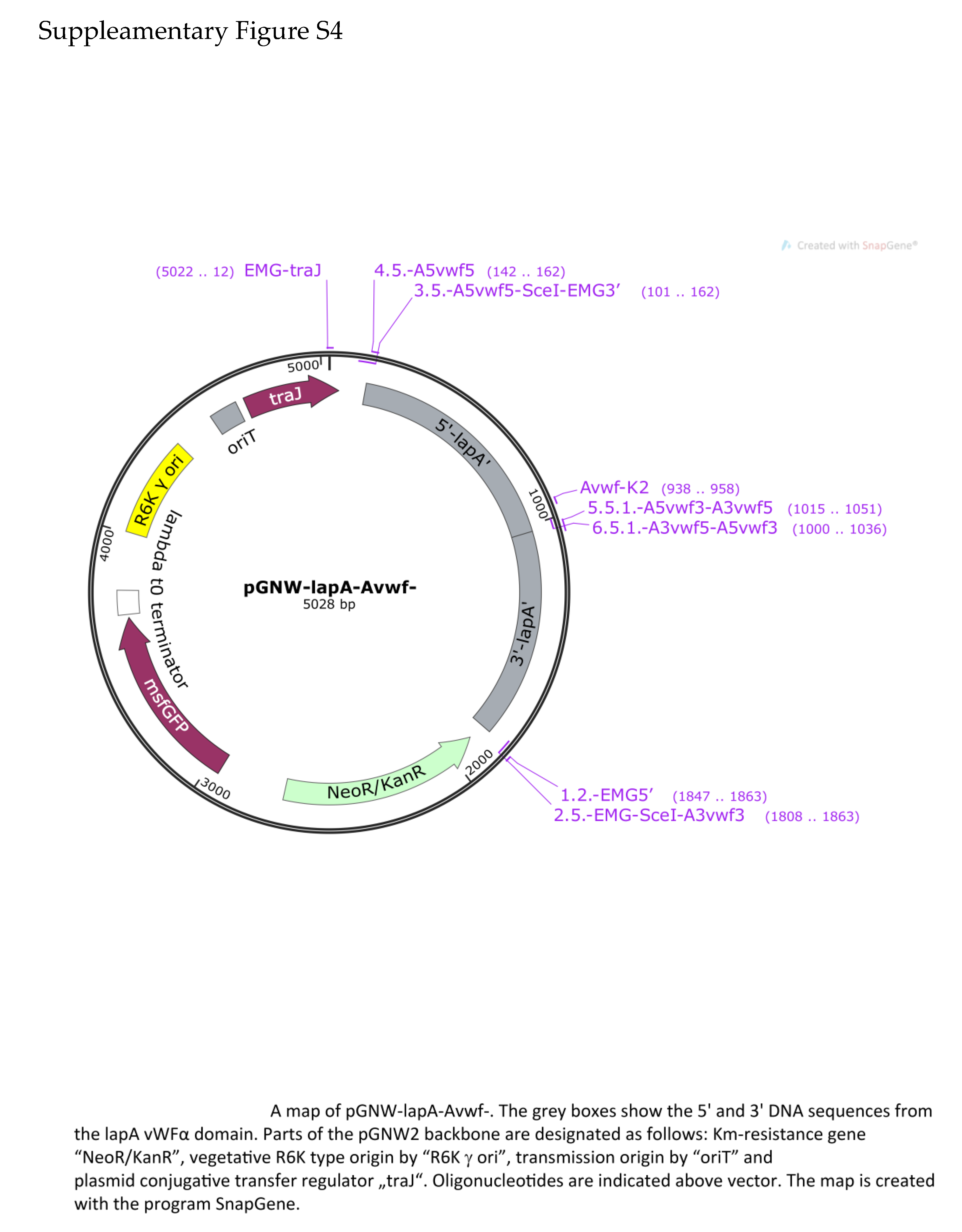

Supplement: Supplementary file 1 [file ijms-23-05898-s001.zip › ijms-1714847-supplementary/Supplementary Figure 4.tif]

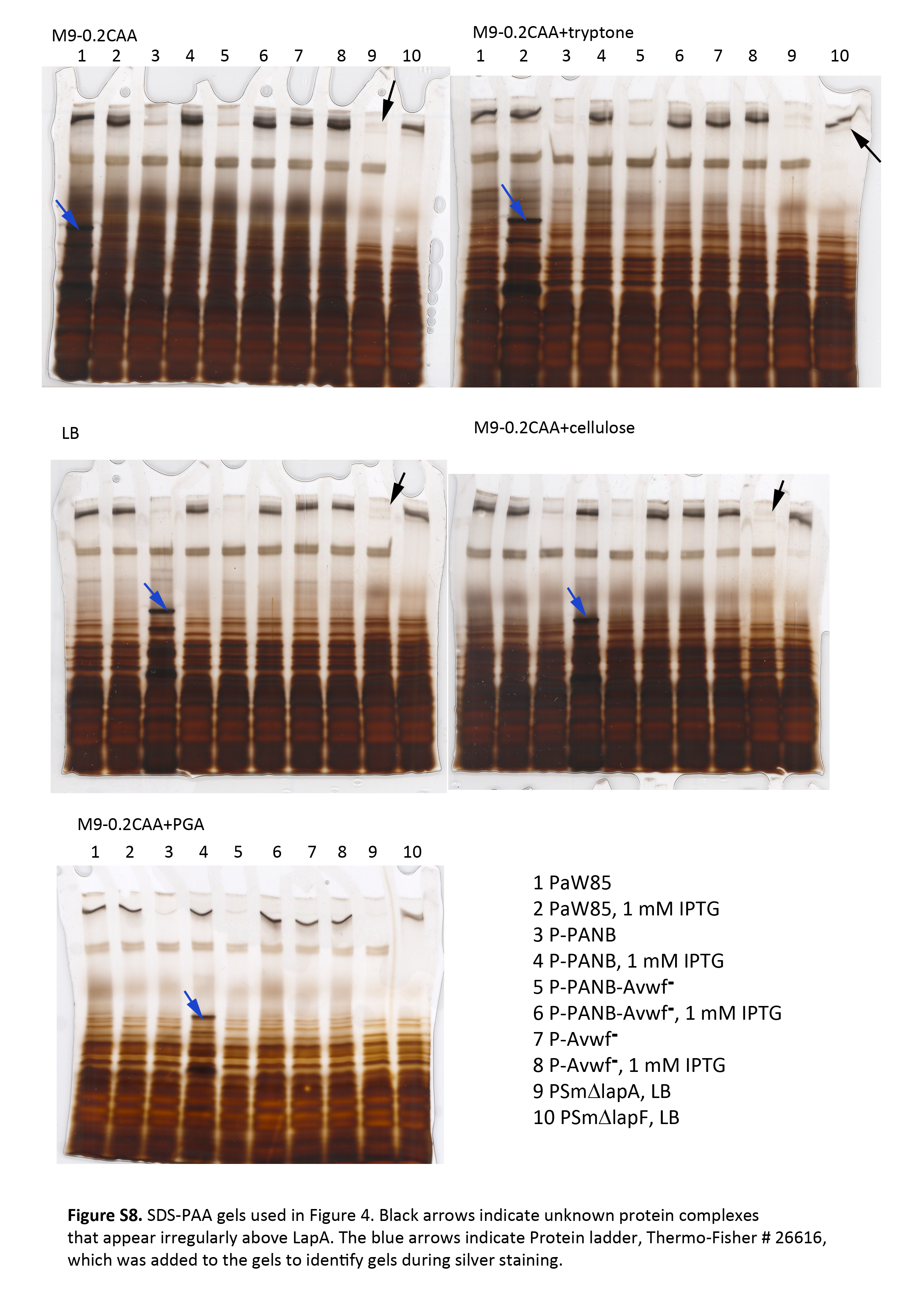

Supplement: Supplementary file 1 [file ijms-23-05898-s001.zip › ijms-1714847-supplementary/Supplementary Figure 8 (SDS-PAAG for Figure 4).tif]
